# Mixed Bartter-Gitelman syndrome: an inbred family with a heterogeneous phenotype expression of a novel variant in the *CLCNKB* gene

**DOI:** 10.1186/2193-1801-3-96

**Published:** 2014-02-18

**Authors:** Amar Al-Shibli, Madinah Yusuf, Issam Abounajab, Patrick J Willems

**Affiliations:** Department of Pediatrics, Tawam Hospital, Al-Ain, P.O. Box: 15258, Kragujevac, United Arab Emirates; Department of Academic Affairs, Tawam Hospital, Al-Ain, United Arab Emirates; GENDIA (GENetic DIAgnostic Network), Antwerp, Belgium

**Keywords:** Bartter syndrome, Gitelman syndrome, Mutation, Phenotype, *CLCNKB* gene

## Abstract

Patients with renal diseases associated with salt-losing tubulopathies categorized as Gitelman and classic form of Bartter syndrome have undergone genetic screening for possible mutation capture in two different genes: *SLC12A3* and *CLCNKB*. Clinical symptoms of these two diseases may overlap.

Bartter syndrome and Gitelman syndrome are autosomal recessive salt-losing tubulopathies with hypokalemia, metabolic alkalosis, hyperreninemia, hyperplasia of the juxtaglomerular apparatus, hyperaldosteronism, and, in some patients, hypomagnesemia.

Here we describe four patients from an inbred family with a novel missense variant in the *CLCNKB* gene. All of patients are asymptomatic; yet they have the typical metabolic abnormality of salt losing tubulopathies. One of those patients had hypomagnesaemia while others not. Clinical and laboratory data of all patients was described. All 4 patients have a homozygous c.490G > T missense variant in exon 5 of the *CLCNKB* gene. This variant alters a glycine into a cysteine on amino acid position 164 of the resulting protein (p.Gly164Cys). The c.490G > T variant is a novel variant not previously described in other patients nor controls. Polyphen analysis predicts the variation to be possibly damaging. Analysis of *SLC12A3* was normal.

Here in we are describing a novel homozygous c.490G > T missense variation was identified in exon 5 of the *CLCNKB* gene was identified in an Emirati patients with a mild manifestation of Bartter - Gitelman syndrome.

## Introduction

Bartter syndrome (BS) and Gitelman syndrome (GS) are autosomal recessive disorders with a characteristic set of metabolic abnormalities (Amirlak and Dawson 2000; Konrad et al. 2000; Naesens et al. 2004). These include hypokalemia, metabolic alkalosis, hyperreninemia, hyperplasia of the juxtaglomerular apparatus (the source of renin in the kidney), hyperaldosteronism, and, in some patients, hypomagnesemia. (Amirlak and Dawson 2000; Naesens et al. 2004).

The main pathogenesis in BS is the defect of NaCl reabsorption in the thick ascending limb of Henle's loop (TALH). (Konrad et al. 2000) Inherited BS can be divided into five subtypes according to the different disease genes involved. Antenatal BS type 1 is due to variants in the *SLC12A1* gene, whereas antenatal BS type II is due to variants in the *KCNJ1* encoding the inward rectifying K + channel (ROMK). Classical Bartter syndrome (type III) is caused by variants in *CLCNKB* encoding the kidney-specific basolateral chloride channel for cBS. Type IV by variants in BSND encoding the β-subunit of the basolateral chloride channel for aBS (type IV), and type V by variants in the calcium-sensing receptors for autosomal dominant hypocalcemia associated with hypokalemia (Amirlak and Dawson 2000; Vargas et al. 2011) Apr as shown in Table 1. In the majority of GS patients, DNA variants are found in the *SLC12A3* gene, which encodes the thiazide- sensitive NaCl co-transporter (NCC). In a small minority of GS patients, variants in the *CLCNKB* gene encoding the chloride channel ClC-Kb have been identified (Vargas et al. 2011) Apr. Konrad et al. (Konrad et al. 2000) and (Jeck et al. 2000) reported that variants in the *CLCNKB* gene not only cause classical Bartter syndrome (type III), but also phenotypes that overlap with either antenatal Bartter syndrome (Types I-II) or Gitelman syndrome. In addition, a pharmacology-based classification and pharmacotype terminology for SLTs were developed and introduced in 2008 (Seyberth 2008) Oct. This classification is based on three major types of salt-losing tubulopathy can be defined: distal convoluted tubule dysfunction (thiazide-like DCT disorders) leading to hypokalemia (currently known as Gitelman or Bartter syndrome type III), the second more-severe condition of polyuric loop dysfunction and furosemide-like loop disorders (often referred to as antenatal Bartter or hyperprostaglandin E syndrome or BS types I and II), and the most-severe condition of combined loop and distal convoluted tubule dysfunction (antenatal Bartter or hyperprostaglandin E syndrome with sensorineural deafness) or BS type IV. (Jeck et al. 2004; Seyberth and Schlingmann 2011) October.

**Table 1:** **Genetics and presentation of Bartter and Gitelman syndromes**

Disorder	Gene affected	Gene product	Clinical presentation
Bartter syndrome type I	*SLC12A1*	NKCC2	Antenatal Bartter syndrome (Hyperprostaglandin E syndrome)
Bartter syndrome type II	*KCNJ1*	ROMK	Antenatal Bartter syndrome
Bartter syndrome type III	*CLCKB*	CLC-Kb	Hypochloremia., mild hypomagnesemia, FTT in infancy
Bartter syndrome type IVA	*BSND*	Barttin (B-subunit of CLC-Ka and CLC-Kb)	Antenatal Bartter syndrome (Hyperprostaglandin E syndrome) and sensorineural deafness
Bartter syndrome type IVB	*CLCNKA* and *CLCNKB*	CLC-Ka and CLC-Kb	Antenatal Bartter syndrome (Hyperprostaglandin E syndrome) and sensorineural deafness
Bartter syndrome type V^•^	*CaSR* gene	CaSR	Bartter syndrome with hypocalcemia
Gitelman syndrome	*SLC12A3*	NCC (thiazide- sensitive NaCl co-transporter).	Hypomagnesemia, hypocalcuria, growth retardation

There are six Bartter syndrome subtypes (I, II, III, IV, IVB, and V) corresponding to six genetic defects. NKCC2: furosemide-sensitive sodium-potassium-2 chloride cotransporter; ROMK: renal outer medullary potassium channel; CLC-Kb: chloride channel Kb; CLC-Ka: chloride channel Ka; CaSR: calcium sensing receptor; NCCT: thiazide-sensitive sodium-chloride cotransporter. *Modified from Seybrerth et al.* (Jeck et al. 2004) Jan.

Here in, we described a case series with mild and heterogeneous phenotype, all had novel mutation in the *CLCNKB* gene.

### Case reports

The first patient (proband1) was a 14 years old girl was presented with prolonged hypokalemia after an attack of acute gastroenteritis. Review of all systems was negative. There was no significant perinatal history, her birth history records revealed full-term by normal spontaneous delivery and normal birth weight of 3,400 grams without antenatal polyhydramnios. There was no history of chronic drug ingestion. She was doing well in school and had normal actively level, normal hearing and vision.

On presentation her height (160 cm) and body weight (58 kg) both were around the 50th percentile. Blood pressure was 114/75 mmHg, heart rate 96 beats/min and respiratory rate 22/min. The remainder of the physical examination was unremarkable. Family history was negative for chronic diseases and sudden death.

The data of biochemical studies are shown in Tables 2 and 3. The most striking findings were hypokalemia (2.4 mmol/l), metabolic alkalosis with arterial PH of 7.49 (7.35-7.45), HCO3 33 mmol/l. (22-28(mmol/L). EKG disclosed a sinus rhythm. Abdominal sonography revealed bilateral normal size kidneys without nephrocalcinosis. Urinalysis revealed negative protein, glucose, leukocytes, and red blood cells. Urine Sodium, Potassium and chloride levels were 199, 62, and 245 mmol/L respectively (all should be less than 20 mmol/L). Average urinary volume was about 1600 ml daily. The trans-tubular potassium gradient (TTKG) was 9.6 (normally it should be less than 3 in the presence of hypokalemia). Serum magnesium (Mg) was tested twice and the value was 0.61 and 0.62 mmol/L respectively, urine Mg was high with Mg/Cr 1.27 mmol/mmol (normal < 0.9) and the fractional excretion of Mg (FeMg) was 3.8 (normal < 2). Serum calcium (Ca) levels were normal, urine Ca/Cr 0.42 mmol/mmol (normal < 0.7). Normal Prostaglandin E2 (PGE2) level (140 ng/24 hrs). Sweat chloride test was normal.

**Table 2:** **Clinical features in all patients**

Clinical feature	Case 1 (index)	Case 2	Case 3	Case 4
Age in years on presentation/follow up	14	8	11	8
Gender	Female	Male	Female	Female
Weight centile on presentation/follow up	75^th^/90^th^	<5^th^/10^th^	15^th^	50^th^
Height centile on presentation/follow up	50^th/^75^th^	30th	10^th^	30^th^
History of polyuria and polydipsia	Negative	Negative	Negative	Negative
Blood pressure	114/75	100/70	110/68	105/68
Nephrocalcinosis/Nephrolithiasis	Negative	Negative	Negative	Negative

**Table 3:** **Laboratory features of all patients**

Laboratory findings	Case 1	Case 2	Case 3	Case 4	Normal ranges
Sodium	130	138	138	137	135-143 (mmol/L)
Potassium	2.4	2.3	2.7	2.3	3.4-4.5 (mmol/L)
Chloride	89	96	96	89	98-106 (mmol/L)
Bicarbonate	33	31	32	32	22-28 (mmol/L)
Serum creatinine	44	30	33	31	27-53 (micromol/L)
Blood urea	3.6	4	2.8	3.9	2.9-7.1 (mmol/L)
Magnesium	0.61	0.92	0.71	0.85	0.74-1.03 (mmol/L)
Renin	216.7	NA	NA	NA	30-40 ng/L (resting)
Aldosterone	324	NA	NA	NA	115-406 ng/L
PGE2	140	70	74	44	400-620 ng/24 hours
Urine Ca/Cr	0.42	0.55	0.06	NA	<0.7 mmol/mmol
Urine Mg/Cr	1.37	0.65	0.28	NA	<0.9 mmol/mmol

**Figure 1:**
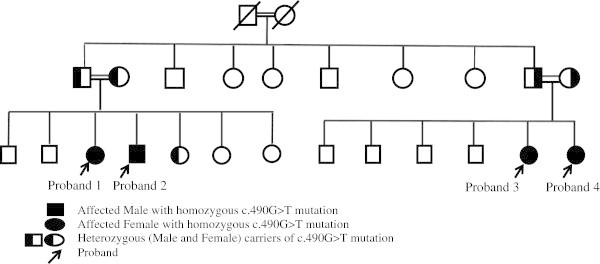
**Family pedigree of the affected patient showing the affected patient proband 1 and other affected family members.** Both parents are cousins and the grand parents were cousins as well. Parents of the two families were having the same mutation in a heterozygous carrier state.

Family was counseled for the screening of other siblings for metabolic abnormalities that was found in another sibling (proband 2) with same hypokalemic metabolic alkalosis (Tables 1 and 2). Two of their cousins were having same manifestation (probands 3 and 4) as explained in the family pedigree (Figure 1). DNA analysis showed genomic DNA was extracted from the peripheral white blood cells. Sequencing of the entire coding region (exons 2-20) and all intron-exon boundaries of *CLCNB* gene was performed. The reference sequence and exon numbering and according to Gene bank accession number NM_000085.2, with the A of the ATG start codon on position 1. A homozygous c.490G > T missense variant was identified in exon 5 of the *CLCNKB* gene. This variant alters a glycine into cystine on amino acid position 164 of the resulting protein (p.Gly164Cys). The c.490G > T variant is a novel variant not previously described in other patients nor controls. Polyphen analysis predicts the variant to be "possibly damaging". All of 5 patients were homozygous for the same variant, whereas their parents were heterozygous for this missense variant. Sequencing of the entire coding region (exons 1-26) and al interons-exons boundaries of the SLC12A3 gene. The reference sequence and exon numbering are according to Genbank accession number NM_000339 with the A of the ATG start codon on position 1 was done and normal results were obtained.

## Discussion

Bartter and Gitelman syndromes are genotypic and phenotypic heterogeneous diseases. Clinical manifestation and laboratory findings might be misleading in proposing diagnosis and managing appropriate treatment. (Jeck et al. 2000).

Our patients were having the typical metabolic abnormalities of BS/GS tubulopathy. Urine Na, K, and Cl were significantly high, TTKG was high in the presence of hypokalemia indicateing renal loss. Their parents and other heterozygous siblings are normal and they don’t have electrolytes abnormalities.

The main difference in the clinical presentation of Bartter and Gitelman Syndromes is explained in Table 4. (Urbanova et al. 2011) The symptoms of Bartter syndrome type III (classical Bartter) often occur in the first 2 years of life, but are usually diagnosed in school age children or even later in adolescence. Patients present with polyuria and polydipsia initially, and growth retardation later becomes evident if no early intervention is done. (Peters et al. 2002) Patients with BS are usually associated with high PGE2 production and hypercalciuria. GS usually has milder presentation with no polyuria and no failure to thrive and usually presented later in life. (Seyberth and Schlingmann 2011) October Our patients showed low PGE2 and low calcium in urine in contrary to patients with BS as they trend to have high urine PGE2 levels and hypercalciuria. (Peters et al. 2002; Brochard et al. 2009)

**Table 4:** **Features differentiating Bartter and Gitelman syndromes**

Features	Classic Bartter syndrome	Gitelman syndrome
Age at onset	Childhood (early)	Childhood or later
Maternal hydramnios	Rare	Absent
Polyuria, polydipsia	Present	Rare
Dehydration	Often present	Absent
Tetany	Rare	Present
Growth retardation	Present	Absent
Urinary calcium	Normal or high	Low
Nephrocalcinosis	Rare	Absent
Serum magnesium	Occasionally low	Low
Urine prostaglandins (PGE2)	High or normal	Normal

Modified from Urbanova et al. (Peters et al. 2002).

In our patient Gitelman was initially diagnosed based on the clinical and laboratory findings and so Genetic analysis for *SLC12A3* gene was done and it was normal; *CLCNKB* gene showed a novel mutation in the exon 5. Co-segregation of this missense variant in an inbred family with 4 affected patients suggest that this variant is pathogenic.

Clinical symptoms and biochemical markers of GS and classic form of Bartter syndrome (type III) may overlap and thus genetic analysis may specify the real cause of symptoms. (Brochard et al. 2009) Our patient had hypomagnesaemia which is due to renal loss based on the high Mg in the urine in the presence of hypomagnesaemia and the high FeMg. Mg supplement was needed for the index patient but not for the others; however other patients may develop hypomagnesaemia in the future as transition phenotypes from classical BS (cBS) to GS have also been described. (Cruz and Castro 2013 Jan).

There is a difference in both clinical and biochemical expression of *CLCNKB* mutations in both GS and type III BS syndromes between patients who share the same mutations suggests. (Briet et al. 2006; Uchida 2000) A modifier effect from genetic and/or environmental factors as it has been often reported in other cases of *CLCNKB* mutation (Dong Yan et al. 2010; Nozu et al. 2007) Sep and other human diseases such as polycystic kidney disease (Fain et al. 2005). However, the genotype–phenotype relation is variable, and mutations in the *CLCNKB* gene may cause overlapping phenotypes of classic/antenatal BS, cBS/GS, and GS (Cruz and Castro 2013; Fain et al. 2005; Peters et al. 2002). Several former studies tried to focus on the correlation between specific DNA mutation and phenotypic clinical outcome. In a study by Coto, many individuals carrying exactly the same mutation coming from unrelated families did not correlate in values of ionic composition in blood and urine. Their clinical symptoms also differed. (Coto et al. 2004) To date, more than 30 *CLCNKB* variants have been reported (Pierre Robitaille et al. 2011; Israel et al. 2003; Fukuyama et al. 2003; Fukuyama et al. 2004; Rodriguez et al. 2005; Yu et al. 2010; Xiumin et al. 2013; Gorgojo et al. 2006; Lee et al. 2012; Toshihiro et al. 2006; Enriquez et al. 2010; Konrad et al. 2000a) in patients with classical BS phenotype, atypical BS or mixed Bartter-Gitelman phenotypes as found in our patients. Our family shows that even patients and even siblings with the same DNA variants could present differences in clinical symptoms, and even mimic a different syndromes. This was the case in a study by Zelikovic, where a large Bedouin family sharing *CLCNKB* variant presented clinical characteristics specific for Gitelman syndrome, on the one side of the spectrum, to classic Bartter syndrome, on the other. (Zelikovic et al. 2003) Therefore, there is an indication for screening the *CLCNKB* gene in those patients with the Gitelman phenotype who do not have variants in the *SLC12A3* gene. (Konrad and Weber 2003).

## Conclusion

Our findings demonstrate intrafamilial phenotypic heterogeneity, namely the presence of Gitelman syndrome and classic Bartter syndrome phenotypes in kindred’s with *CLCNKB* c.490G > T mutation.

## Consent

Written informed consent was obtained from the patients' parents for the publication of this report and any accompanying images.
